# An improved YOLO v4 used for grape detection in unstructured environment

**DOI:** 10.3389/fpls.2023.1209910

**Published:** 2023-07-13

**Authors:** Canzhi Guo, Shiwu Zheng, Guanggui Cheng, Yue Zhang, Jianning Ding

**Affiliations:** ^1^ Institute of Intelligent Flexible Mechatronics, Jiangsu University, Zhenjiang, China; ^2^ Jiangsu Collaborative Innovation Center of Photovoltaic Science and Engineering, Changzhou, China; ^3^ School of Mechanical Engineering, Yangzhou University, Yangzhou, China

**Keywords:** harvesting robot 1, picking robot 2, YOLO 3, object detection 4, attention mechanism 5, unstructured environment 6

## Abstract

Visual recognition is the most critical function of a harvesting robot, and the accuracy of the harvesting action is based on the performance of visual recognition. However, unstructured environment, such as severe occlusion, fruits overlap, illumination changes, complex backgrounds, and even heavy fog weather, pose series of serious challenges to the detection accuracy of the recognition algorithm. Hence, this paper proposes an improved YOLO v4 model, called YOLO v4+, to cope with the challenges brought by unstructured environment. The output of each Resblock_body in the backbone is processed using a simple, parameterless attention mechanism for full dimensional refinement of extracted features. Further, in order to alleviate the problem of feature information loss, a multi scale feature fusion module with fusion weight and jump connection structure was pro-posed. In addition, the focal loss function is adopted and the hyperparameters α, γ are adjusted to 0.75 and 2. The experimental results show that the average precision of the YOLO v4+ model is 94.25% and the F1 score is 93%, which is 3.35% and 3% higher than the original YOLO v4 respectively. Compared with several state-of-the-art detection models, YOLO v4+ not only has the highest comprehensive ability, but also has better generalization ability. Selecting the corresponding augmentation method for specific working condition can greatly improve the model detection accuracy. Applying the proposed method to harvesting robots may enhance the applicability and robustness of the robotic system.

## 1 Introduction

In recent years, with the increase of population, the demand for fruits and vegeTables has increased. However, in order to save costs, the harvest of fruits and vegeTables mainly relies on untrained workers rather than skilled farmers, which inevitably leads to damage of fruits and vegeTables. Therefore, traditional manual picking of fruits and vegeTables not only consumes essential labor resources (Lawal, 2021), but also reduces efficiency. As most countries in the world are gradually stepping into the aging society, the problem of labor shortage will follow. It is vital to replace traditional agriculture with intelligent agricultural relying on mechanical automation. Robots with human visual perception capabilities provide the possibility for intelligent automated harvesting (Li et al., 2021). Improving the accuracy of fruits and vegeTables recognition has become a crucial research field (Silwal et al., 2017).

In order to realize the vision function of harvesting robots, a great amount of researchers have proposed various target detection algorithms based on the contour, color, texture and other characteristics of fruits and vegeTables. Lin (Lin et al., 2020) proposed a probabilistic Hough transform to aggregate shape segments matched based on contour information to obtain candidate fruits. This method is not scale-invariant and fails to detect fruits with large scale changes, which is caused by using the difference of scale variables to determine the probability value. Chaivivatrakul (Chaivivatrakul and Dailey, 2014) proposed a method to analyze the texture information of green fruits by using different combinations of Scale-invariant Feature Transform (SIFT), Speeded-Up Robust Feature (SURF), Oriented FAST and Rotated BRIEF (ORB), etc. According to the matching of extracted points of interest, the fruit detection accuracy reached more than 90%, but detection effect is easily affected by strong sunlight and occlusion, which leads to the low robustness of system. Payne (Payne et al., 2013) proposed a night detection algorithm based on RGB and YCbCr color features and texture segmentation of adjacent pixels variability. In order to eliminate the problem of the algorithm overly relying on color features, the hessian filter was used to remove the texture features of leaves, trunk and stems. Even though this algorithm only has an error rate of 10.6%, it needs to adjust the corresponding filter settings according to different in-field systems, which does not have high compatibility.

The above-mentioned traditional algorithms based on contour, color, and texture information generally suffer from poor stability and are difficult to apply to unstructured environment, especially in dynamic weather conditions. With the continuous development of machine learning, especially deep convolutional neural networks, object detection algorithms have been widely used in harvesting robots to identify and locate fruits and vegeTables in recent years (Darwin et al., 2021) (Cecotti et al., 2020). Deep learning can effectively reduce the impact of light fluctuations, occlusion of branches and leaves, and overlap of fruits when processing fruit detection tasks (Yin et al., 2021).

Generally, object detection algorithms in deep learning can be divided into two categories, one is the two-stage object detection models represented by Fast R-CNN (Girshick, 2015), Faster R-CNN (Ren et al., 2017), Cascade R-CNN (Cai and Vasconcelos, 2018), and Libra R-CNN (Pang et al., 2019). This type of algorithm has a special region proposal network (RPN) for candidate target extraction, and then input the pre-extracted region of interest (RoI) into convolutional neural network (CNN) for regression classification prediction. The other type is the one-stage network that uses a single CNN network to perform regression prediction on the input image, which predicts the target category while generating the bounding box for location. Representative networks of this type are Single Shot Multi-Box Detector (SSD) (Liu et al., 2016), RetinaNet (Lin et al., 2017), EfficientDet (Tan et al., 2020) and You Only Look Once v4 (YOLO v4) (Bochkovskiy et al., 2020. In the one-stage object detection models, target positioning and classification are completed at the same time, so their advantage is faster detection speed. However, due to the lack of high-quality candidate anchors extracted by RPN, the detection accuracy of one-stage network is not as good as that of two-stage network. In addition, there are detection algorithms different from the above anchor-base network, such as YOLO v8 (Jocher, 2023), CornerNet (Law and Deng, 2020), CenterNet (Zhou et al., 2019), FCOS (Tian et al., 2019), SAPD (Zhu et al., 2020). These anchor-free detection algorithms get rid of the huge amount of anchors generated by model predictions and complex hyperparameters. These models represent the highest performance of object detection algorithms and often serve as the basis for many optimized models.

Many researchers apply deep learning algorithms to the identification and location of fruits and vegeTables. Song developed a Faster R-CNN model implemented by VGG16 to detect kiwifruit in the natural field, which can work stably during the day and night (Song et al., 2019). The mean Average Precision (mAP) of this detector can reach 87.61%, and it takes 0.347s to detect each picture using NVIDIA TITAN XP 6GB GPU, but there are cases of missed or wrong detection of dense fruits. Cai designed an improved SSD algorithm, which uses soft Non-Maximum Suppression (NMS) to filter out suiTable bounding boxes (Cai et al., 2020). In order to obtain sTable and predicTable gradient of algorithm, batch normalization was used to initialize the de-random training model. The average precision of this model reached 92.4%, and the detection speed using NVIDIA Titan RTX1070 GPU was 36 frames per second (FPS). Moreira compared the detection ability of YOLO v4 and SSD for tomatoes and found that YOLO v4 performed better with an F1 score of 85.81% (Moreira et al., 2022). Su proposed a lightweight model based on YOLO (Su et al., 2022). The backbone of the model adopts Uniform, and the neck adopts a Bi-PAN structure, which combines Bi-FPN and path aggregation network (PAN). NMS is replaced with R-NMS to filter out redundant boundary frames. This method uses NVIDIA RTX 2080Ti GPU with a mAP of 87.7%, F1 score of 83.1%, and FPS of 46 in WGISD. Li proposed an L-YOLO model based on YOLOv3, which replaced DarkNet with the SE_ResGNet34 structure (Li et al., 2021). Squeeze-and-Excitation network (SENet) and grouped convolutions are applied to SE_ResGNet34, and the new backbone can achieve more accurate detection performance while requiring fewer parameters. That is why the average accuracy and detection speed of L-YOLO using NVIDIA Tesla V100 GPU reached 96% and 106 FPS, respectively. However, this model is difficult to detect tiny lemon, especially fruits largely obscured and overlapped. Zhang reduced the width and depth of YOLO v5s, and achieved 344.83 FPS using NVIDIA RTX 2080Ti GPU, but reduced accuracy (Zhang et al., 2022). Pinheiro created and published two datasets for grape detection and classification, and applied YOLO v5, YOLO v7, and YOLO R models to grape detection. The results showed that for identifying bunches, the YOLOv7 model presenting the best performance, achieving 98% of precision, 90% of recall, 94% of F1-score and 77% mAP, and for the classification task, YOLOv5 being the best one, achieving 72% of mAP. (Pinheiro et al., 2023). Meanwhile, Sozzi also compared the performance of six different YOLO series models in detecting green grapes and found that the standard version of YOLO v4 was superior to YOLO v5-x in terms of accuracy and speed, while the Tiny version of YOLO v4 best considered both accuracy and speed (Sozzi et al., 2022) (Nguyen et al., 2023).

According to the above review, most of the detection models are limited by severe occlusion, fruits overlap and illumination fluctuations. In order to improve the performance of the model in response to complex environmental conditions, this paper proposes an improved detection model based on YOLO v4 model, which has a trade-off between average precision and detection speed. SimAM attention mechanism (Yang et al., 2021) and improved feature fusion structure are applied to this model. Moreover, the adapted focal loss function is used in model training. These optimizations help YOLO v4 algorithm to make more reasonable use of computing resources and have stronger feature propagation performance when processing grape images in unstructured environment.

The remainder of this paper is organized as follows: Section 2 describes the construction of improved YOLO v4 model, including the introduction of SimAM block and optimized feature fusion module. Section 3 describes the hardware equipment, hyperparameter settings, image enhancement methods, evaluation indicators, anchor boxes, and loss functions used in the experiments. Section 4 describes the relevant experiments of the detection performance of the proposed model in unstructured environment and discusses the experimental results. Section 5 provides conclusions drawn based on the results.

## 2 Methodologies

### 2.1 YOLO v4 model

YOLO v4 is a detection model optimized by a series of state-of-the-art (SOTA) tricks based on YOLO v3. The architecture of the YOLO family can be divided into three parts: backbone, neck, head. In terms of architecture, the two parts are optimized except for the head of the model. The backbone of YOLO v4 uses CSPDarkNet 53, which replaces the original DarkNet 53 for feature extraction. The feature fusion module in the neck adopts a spatial pyramid pooling (SPP) structure and PAN.

Moreover, the tricks selected in the training process include Mosaic data augmentation, Label smoothing, CloU regression loss, Cosine annealing scheduler, etc. Another improvement is to replace the activation function from LeakyReLU to Mish, which has smoother gradient and better generalization (Misra, 2019). The Mish activation function is defined as follows:


(1) $$Mish=x*\text{tanh}\left[\ln\left(1+e^{x}\right)\right],$$

The SOTA tricks mentioned above are part of the optimization methods adopted in YOLO v4. As one of the most widely used detection models, YOLO v4 takes into account the speed and accuracy of detection. Therefore, the optimization model proposed in this paper is a further improvement of YOLO v4.

### 2.2 SimAM attention mechanism

In computer vision, the attention mechanism redistributes computing resources according to the importance of features. The weighted feature layer has more useful information about the target, which can increase model performance. However, since the essence of existing attention mechanisms is convolution operations, the number of parameters of the model increases (Woo et al., 2018), which imposes a burden on detection speed. Fortunately, the SimAM, a simple and parameter-free attention module, can alleviate the above burden. The SimAM attention mechanism is inspired by the way human brain neurons are activated, and its main idea is the energy function based on neuroscience theory. After simplification and regularization, the final energy function is shown as follows:


(2) $$e_{t}\left(\omega_{t},b_{t},x_{i}\right)=\frac{1}{M-1}\sum_{i=1}^{M-1}{\left(-1-\left(\omega_{t}x_{i}+b_{t}\right)\right)}^{2}+{\left(1-\left(\omega_{t}t+b_{t}\right)\right)}^{2}+\lambda \omega_{t}{\;}^{2},$$

where $x_{i}$ represents non-target neurons in one channel of the input feature map; *I* is index over spatial dimension and *M* represents the number of pixels on a single channel; $\omega_{t}$ and $b_{t}$ respectively represent weight and bias of the transformation; λ is the hyperparameter which is set to $10^{-4}$ .

Studies have shown that our brains use spatial attention and channel attention at the same time when processing visual information (Carrasco, 2011). Therefore, the SimAM attention mechanism evolved by simulating neuron activity can estimate 3D weights for feature maps. According to the spatial suppress effect of activated neurons (Webb et al., 2005), these full 3D weights will be assigned to each neuron, which can strengthen target neurons and suppress other neurons. In order to find these neurons with spatial suppress effect, the linear separability between an interesting neuron and uninteresting neurons is obtained by minimizing the equation 2. After a series of iterative solvers like Stochastic Gradient Descent (SGD) processing, a fast convergent solution is calculated. And, a reasonable assumption that all pixels in a single channel follow the same contribution is applied to this solution. Finally, an equation for computing the minimum energy is derived and shown below:


(3) $$e_{t}^{*}=\frac{4\left({\hat{\sigma }}^{2}+\lambda \right)}{{\left(t-\hat{\mu }\right)}^{2}+2{\hat{\sigma }}^{2}+2\lambda }\;,$$

where $\hat{\mu }$ and ${\hat{\sigma }}^{2}$ represent mean and variance of all neurons in a single channel of the feature map. Equation 3 indicates that the uniqueness of the pixel becomes more obvious as the minimum energy $e_{t}^{*}$ decrease. In other words, the importance of the pixel is proportional to the minimum energy $1/e_{t}^{*}$ . In order to achieve the weighted feature map, Sigmoid activation function is performed on $1/e_{t}^{*}$ , and then the activated energy is multiplied by the input feature map *x_i_*. This refinement phase is shown as follows:


(4) $$\tilde{X}=Sigmoid\left(\frac{1}{E}\right)*X\;,$$

The SimAM attention block is different from traditional attention mechanisms in that there are no layers of convolution, pooling and full connection. Instead, SimAM block derives an energy function to estimate the importance of each feature point. Because of this, SimAM will be used to optimize YOLO v4.

### 2.3 Improved feature fusion structure

The feature fusion module is a structure that combines low-resolution feature maps with rich semantic information and high-resolution feature maps with rich positioning information. This multi-scale fusion method is essential to improve model detection performance (Hu et al., 2021). In order to strengthen the feature propagation ability of feature map, we refer to the feature fusion module named Bi-FPN proposed in EfficientDet to optimize the PAN structure used by YOLO v4. The improved PAN module can provide YOLOhead with feature maps that carry efficient target information, so that the model can perceive more detailed features.

**Figure 1:**
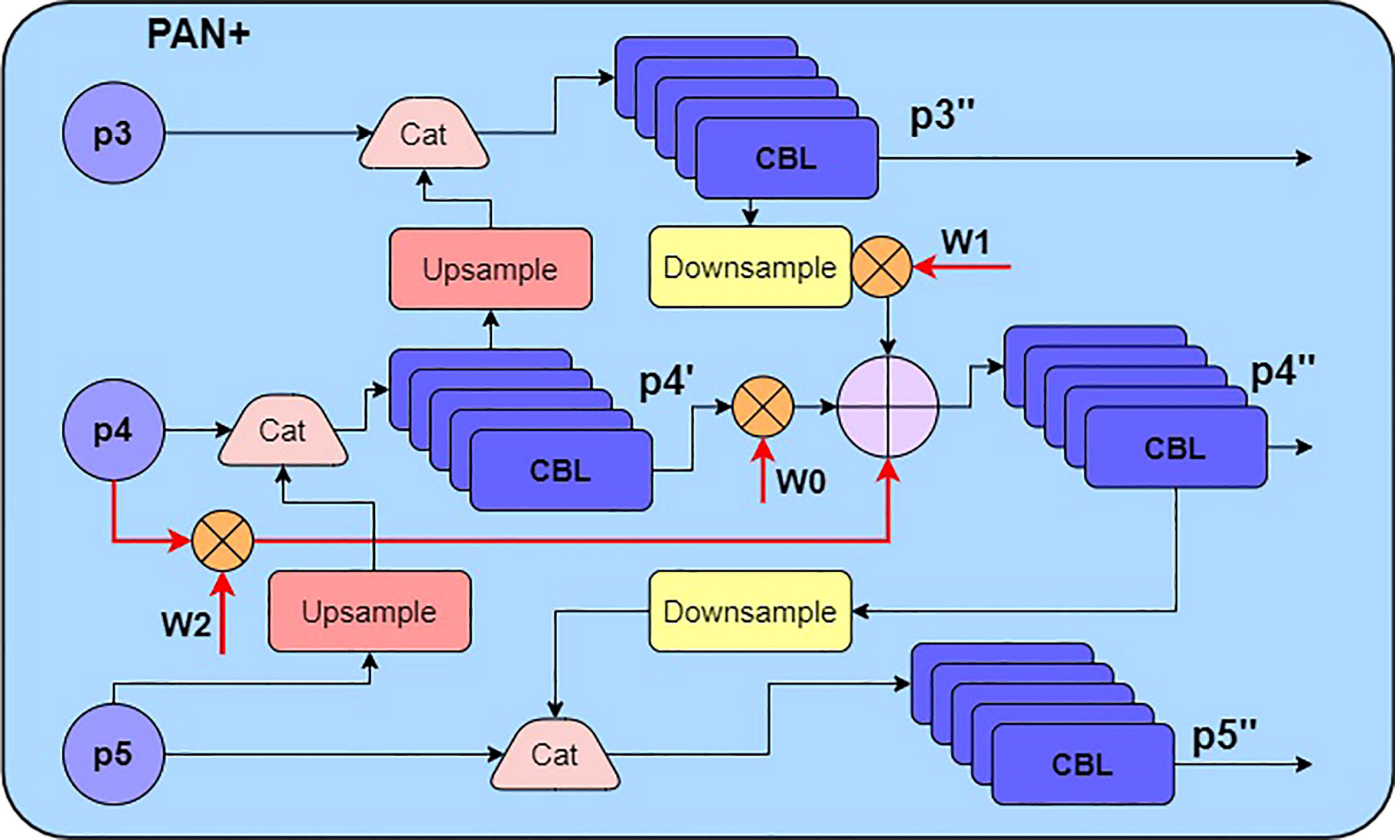
The structure of the improved PAN module.


Paying attention to the connection structure of Bi-FPN (Tan et al., 2020), it adds extra fusion paths to the simplified PAN, which removes those less contribution nodes with only one input path. After Bi-FPN has completed the bidirectional feature pyramid fusion, it also connects the final output and the input feature map of the same resolution. In order to further enrich the target information contained in the feature map, the Bi-FPN structure assigns a simple weight to each processed feature map. The additional fusion process taking P4 in **Figure 1** as an example is shown as follows:

(5) $$P_{4}^{\text{""}}=Conv\left(w_{0}*P_{4}^{"}+w_{1}*Downsample\left(P_{3}^{"}\right)+w_{2}*P_{4}\right)\;,$$

where $w_{0}$ , $w_{1}$ , $w_{2}$ are balance weights; $P_{i}$ , $P_{i}^{"}$ , $P_{i}^{\text{""}}$ represent the input, middle, output feature map, respectively.

**Figure 2:**
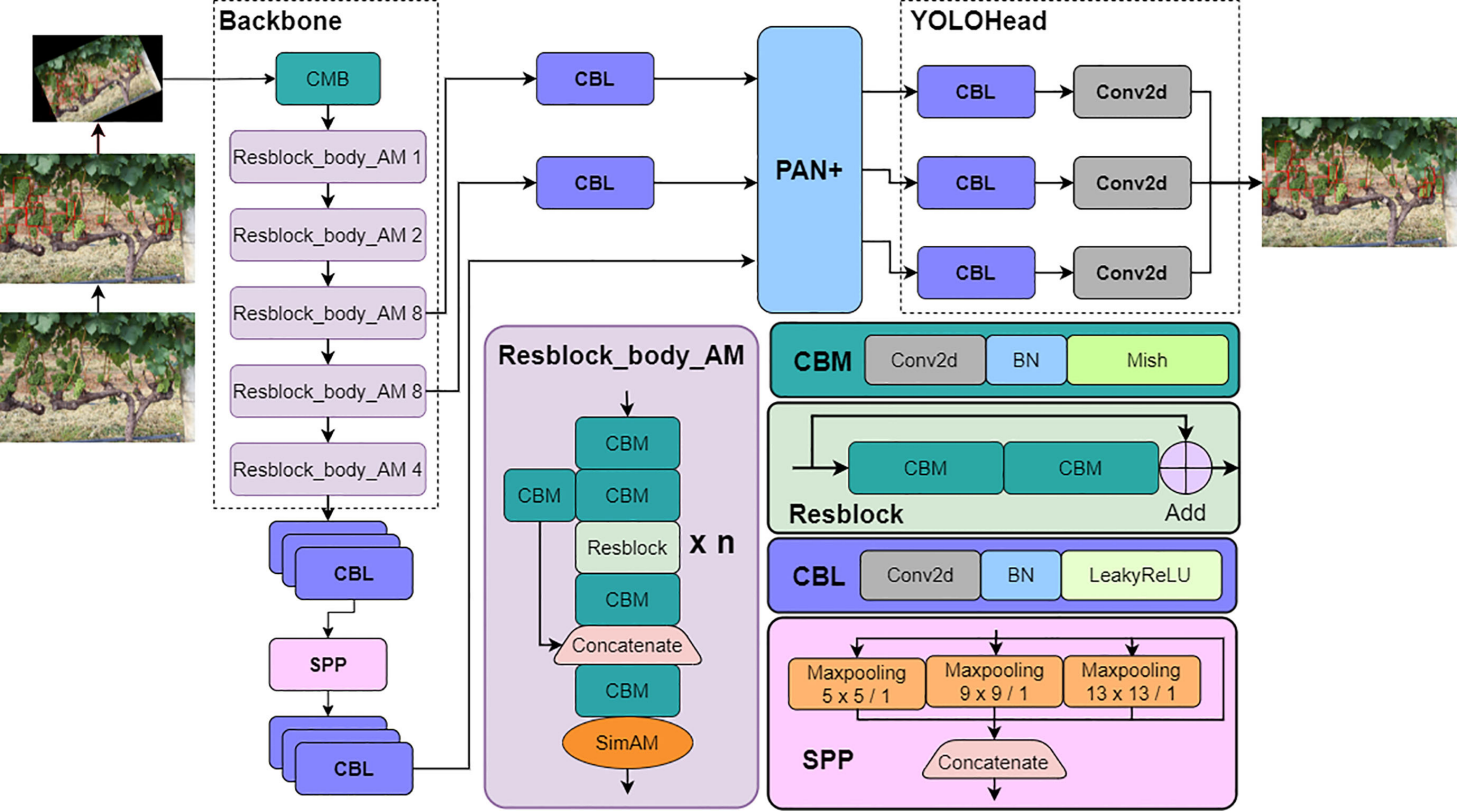
The architecture of YOLO v4+ detection model.

To integrate extracted features maps more adequately, an improved PAN fusion module is proposed in this paper, which uses a convolution group consisting of 3×3 and 1 × 1 standard convolutions instead of the depthwise separable convolution. Considering the cost of video memory and computations, YOLOv4+ repeats fusion module once, which is different from Bi-FPN repeating the same module multiple times. In addition, the fast normalized fusion method (Tan et al., 2020) is used to generate balance weights in equation 5. The improved PAN module structure is shown in **Figure 1**, where CBL block explained in **Figure 2**.

Three feature maps $P_{3},\;P_{4},\;P_{5}$ of different scales are extracted by backbone and SPP module, and used as the input of improved PAN feature fusion module. With more convolution transformations, the resolution of feature map *P*_5_ is reduced to 19×19. The purpose of applying up-sampling transformation to *P*_5_ is to splice *P*_5_ and *P*_4_ which scale is 38×38. After that, five consecutive convolutions adjust the channel information of the spliced feature map, and then $P_{4}^{"}$ is obtained. The fusion of *P*_3_ and *P*_4_ is consistent with the above process. Importantly, $P_{3}^{\text{""}}$ , the output feature layer of 76×76 scale, is multiplied by the weight $w_{1}$ after down-sampling. And the operation result is added with *P*_4_ and $P_{4}^{"}$ , which are assigned to weights of $w_{2}$ and $w_{0}$ respectively. Similarly, the summed feature map is processed by the convolution group to obtain $P_{4}^{\text{""}}$ , which is the output of 38x38 scale. Finally, the output feature map $P_{5}^{\text{""}}$ at the 19×19 scale is obtained by splice down-sampled $P_{4}^{\text{""}}$ and *P*_5_.

### 2.4 Proposed detection model

In view of the full-dimensional correction capability of SimAM block on feature map and the effective acquisition capability of weighted feature fusion module for target feature, YOLO v4+ model with SAM-CSPDarkNet as Backbone and PAN+ as part of Neck was proposed. The model architecture is shown in **Figure 2**.

**Table 1** further describes the extraction process of the backbone, which specifically shows the changes in the shape of feature maps and the size of the convolution filters.

**Table 1:** The parameters of backbone in YOLO v4+.

Type		Size	Shape	Type		Size	Shape
	CBM	3 × 3	6082 × 32	_body _AM	CBM	1 × 1	
Stage 1: Resblock _body _AM	CBM	3 × 3/2	3042 × 64		CSP	1 × 1	762 × 256
	CBM	1 × 1			SimAM	—	
	1 × Resblock	—		Stage 4: Resblock _body _AM	CBM	3 × 3/2	382 × 512
	CBM	1 × 1			CBM	1 × 1	382 × 256
	CSP	1 × 1			8 × Resblock	—	
	SimAM	—			CBM	1 × 1	
Stage 2: Resblock _body _AM	CBM	3 × 3/2	1522 × 128		CSP	1 × 1	382 × 512
	CBM	1 × 1	1522 × 64		SimAM	—	
	2 × Resblock	—		Stage 5: Resblock _body _AM	CBM	3 × 3/2	192 × 1024
	CBM	1 × 1			CBM	1 × 1	192 × 512
	CSP	1 × 1	1522 × 128		4 × Resblock	—	
	SimAM	—			CBM	1 × 1	
Stage 3: Resblock	CBM	3 × 3/2	762 × 256		CSP	1 × 1	192 × 1024
	CBM	1 × 1	762 × 128		SimAM	—	
	8 × Resblock	—					

As shown in **Figure 2**, after Resblock_body_AM module extracts interesting feature pixels, the SimAM attention mechanism calculates the energy density to generate 3D weights for feature maps. Through this process, the feature extraction performance of the backbone can be optimized. Then, the receptive field of the feature map processed by the SPP module is significantly expanded. However, as the input image is repeatedly extracted by Resblock_body_AM, the feature map is gradually shrinking. Because of this, the location information contained in the feature map is inevitably lost, which greatly challenges the accuracy of model detection. To solve above problem, the feature maps extracted in stage 3, stage 4 and the feature map processed by SPP module are input into the improved PAN+ module for feature fusion. Finally, YOLOhead performs regression prediction on the target category and location on three feature maps with scales of 76 × 76, 38 × 38, and 19 × 19.

The processings of the five Resblock_body_AM stages are similar, but the difference lies in the number of times that Resblock is repeated. The first convolutional CBM block with the kernel size of 3 × 3 is used to lengthen the channel of the input image. In order to filter out the noise information in the feature map and enrich the feature information in the channel dimension, the feature map is continuously compressed by CBM blocks, which has a convolution with kernel size of 3×3 and stride of 2, and the size of the feature map shrinks while the channel dimension increases.

## 3 Experimental process and analysis

### 3.1 Experimental setup

This YOLO v4+ model is implemented in the development framework of Pytorch 1.8.1 that supports CUDA 11.0 in Python 3.8.5 environment. A computer equipped with Intel@CoreTMi9-10900K@5.1GHz CPU and NVIDIA RTX 2080Ti GPU which has 11GB memory is used to train and evaluate YOLO v4+ model. The compilation script runs on Ubuntu 18.04.

In order to better match the hardware equipment and improve the detection effect, the model is trained in two stages. In the first stage, the parameters of backbone are frozen, and batch size set to 8, initial learning rate to 0.001, train epochs to 30. In the second stage, all parameters of model are trained, and the batch size set to 2, initial learning rate to 0.0001, train epochs to 10. A prerequisite for the two-stage training strategy is that we employ transfer learning. Throughout the whole training process, cosine annealing scheduler and label smoothing method which hyperparameter is 0.005 are adapted. In addition, the images used for model training adopt the random scale strategy, which magnification factor ranges from 0.7 to 0.9.

### 3.2 Dataset pre-processing

In this study, Wine Grape Instance Segmentation Dataset (WGISD) (Santos et al., 2019) is selected as the dataset of YOLO v4+. The dataset consists of five categories of grape, with a total of 300 images containing 4432 grape clusters identified by bounding boxes. Images were resized to width of 2048 pixels in order to retain more detailed information (Wu et al., 2020). The annotated “YOLO” format in WGISD is converted to “Pascal VOC” format through programming, which has higher compatibility, because the annotation can be applied to more supervised learning detection models. The specific operation is to convert the category index and the center coordinates, width, and height of the bounding box in “YOLO” format into corresponding category names and upper left, lower right coordinates of the bounding box, respectively, and then store them in XML tags in “Pascal VOC” format.

The advantages of image pre-processing are to enrich the information contained in the dataset and improve the robustness of the model. The data augmentation methods used in this study, which enlarges the dataset by 6 times, include brightness transformation, blur processing, affine transformation, mirror transformation, and raindrop processing, and the completed dataset is shown in **Table 2**.

**Table 2:** The number of images generated by data augmentation methods.

Variety	Original data	Brightness	Gaussian Blur	Affine Mapping	Mirroring	Raindrop	Bounding boxes
Chardonnay	65	65	65	65	65	65	**840**
Cabernet Franc	65	65	65	65	65	65	**1069**
Cabernet Sauvignon	57	57	57	57	57	57	**643**
Sauvignon Blanc	65	65	65	65	65	65	**1317**
Syrah	48	48	48	48	48	48	**563**
**Total**	**300**	**300**	**300**	**300**	**300**	**300**	**4432**

**Figure 3:**
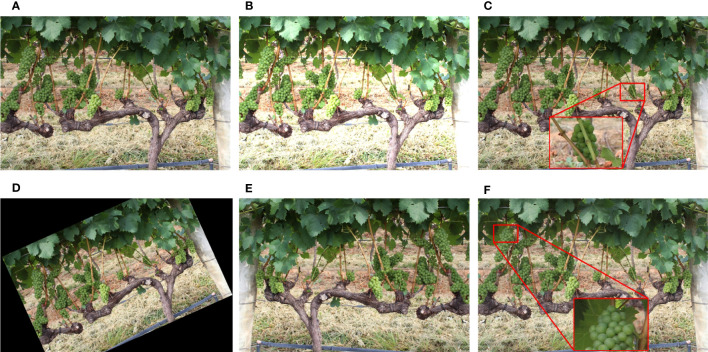
Image augmentation methods: **(A)** original image, **(B)** brightness transformation, **(C)** blur processing, **(D)** affine transformation, **(E)** mirror transformation, **(F)** raindrop processing.

The dataset is augmented as shown in **Figure 3**. Since the angle and intensity of the illumination are constantly changing throughout the day, we have performed a brightness transformation on the dataset. The purpose of Gaussian blur processing is to reduce the impact of dust on the camera lens and inappropriate focus shots. For further expand the dataset, affine transformation and mirror transformation are used. To simulate a rainy day, the original image is performed on raindrop processing to enhance the robustness of the model.

### 3.3 Evaluation indicators

FPS (frames per second), F1 score, AP (average precision) are selected as indicators to evaluate the quality of detection models. FPS is used to measure the speed of model detection. The key parameters for calculating F1 score and AP are P (precision) and R (Recall), which are defined as follows:


(6) $$P=\frac{TP}{TP+FP}\;;R=\frac{TP}{TP+FN}\;,$$

where TP, FP, FN represent positive samples that are predicted correctly, negative samples that are predicted incorrectly, positive samples that are predicted incorrectly respectively. According to the definition, R represents the search ability of the model for objects.

F1 score is a comprehensive consideration of P and R as they may contradict. The average precision of each class is called AP, which value can be expressed by the area under the P-R curve. Their calculation equations are as follows:


(7) $$F_{1}score=2*\frac{P*R}{P+R}\;,$$

(8) $$AP=\int_{0}^{1}PdR\;,$$

### 3.4 Anchor boxes

**Figure 4:**
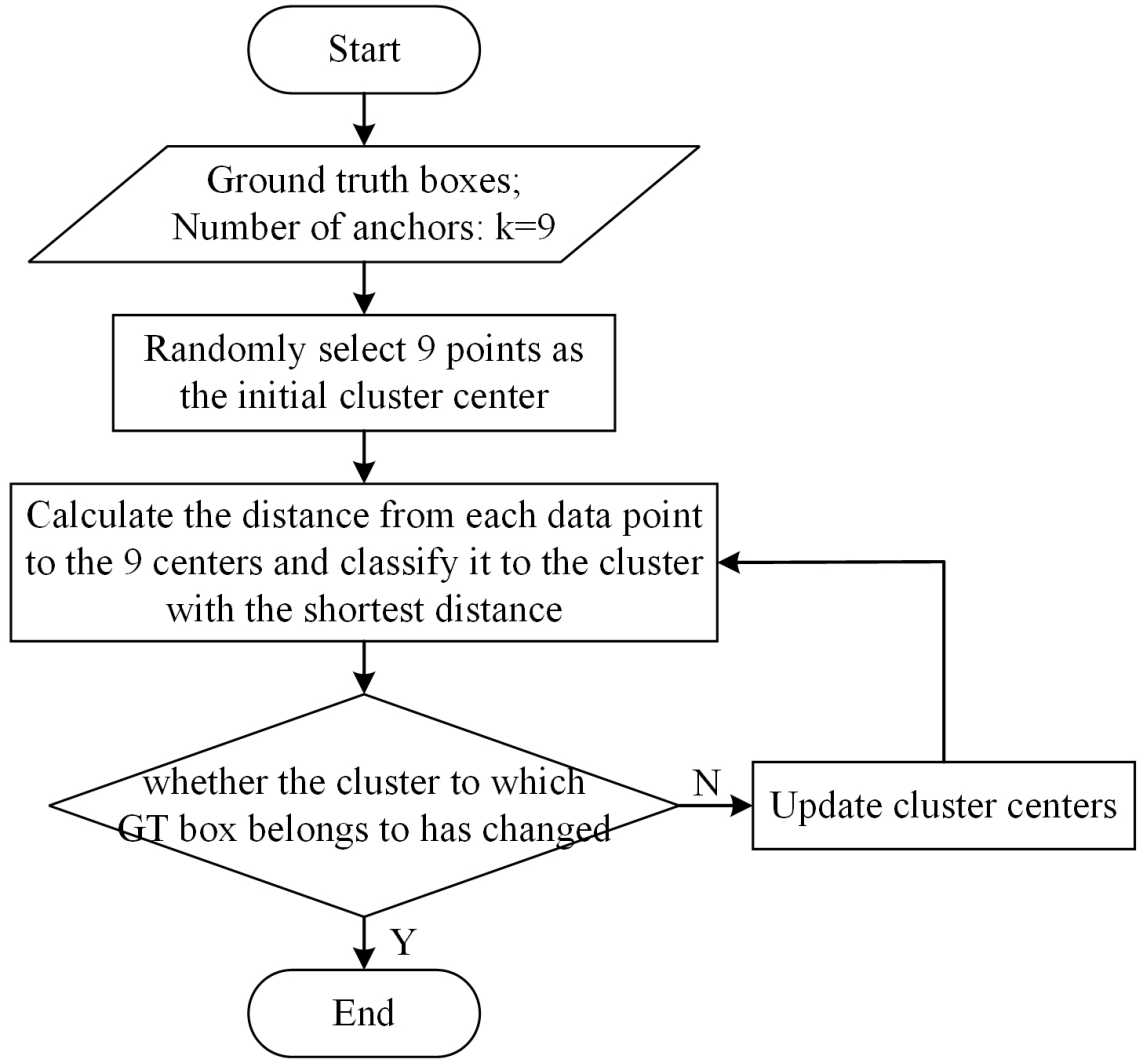
The flowchart of the K-means clustering algorithm.

After feature extraction and fusion through the YOLO v4+ model, three feature maps of different scales (76×76, 38×38, 19×19) will be imported into YOLOhead to predict the target. The feature map of each scale will use three corresponding anchor boxes, and the sizes of these anchor boxes are obtained by the following method:

The target category detected by this model is now particular, the K-means clustering algorithm is used to generate 9 anchor boxes which are suiTable for the grape dataset. The flowchart of the K-means algorithm is shown in **Figure 4**. The information input to the algorithm is the width and height of ground truth (GT) Boxes and the number of clusters. Firstly, 9 data points are randomly selected as the initial cluster center. Secondly, the program calculates the distance from each data point to 9 cluster centers and divide them into the cluster with the shortest distance. Finally, judge whether the cluster of each point has changed, if it changes, update the cluster center according to the average distance of all data points in the same cluster, otherwise output 9 cluster centers. The sizes of the 9 anchors clustering by the K-mean algorithm is shown in **Table 3**.

**Table 3:** The width and height of anchor boxes and clustering accuracy.

Anchor	Width	Height
1	21.35	34.31
2	28.03	52.07
3	29.71	82.07
4	39.81	62.96
5	39.88	105.56
6	48.79	146.02
7	54.89	77.41
8	65.68	208.82
9	69.43	114.00
**Accuracy**	**78.66%**	

### 3.5 Loss function

Loss function is used to calculate the deviation between the ground true value and the predicted value. According to loss value, the weights are updated in the back propagation in CNN, so that neural network can fits an approximate non-linear model that meets the established task. The Binary Cross Entropy (BCE) optimized by focal loss function (Lin et al., 2017), and Complete Intersection over Union (CIoU) loss function (Zheng et al., 2020) are adopted in YOLO v4+. The total loss function used for model training is shown as follows:


(9) $$Loss=\frac{\sum_{0}^{2}\lambda_{conf}L_{fl}\left(l_{conf}\right)+\lambda_{cls}L_{fl}\left(l_{cls}\right)+\lambda_{loc}l_{loc}}{batch\_size}\;,$$

where $\lambda_{conf}$ , $\lambda_{cls}$ , $\lambda_{loc}$ are the balance coefficients of confidence, classification and location loss respectively. In this study, $\lambda_{conf}=\lambda_{cls}=\lambda_{loc}=1$ are selected. The confidence loss $l_{conf}$ is defined as follows:


(10) $$l_{conf}=-\sum_{i=0}^{s^{2}}\sum_{j=0}^{B}1_{ij}^{obj}\left[c_{ij}\log\left(\hat{c_{ij}}\right)+\left(1-c_{ij}\right)\log\left(1-\hat{c_{ij}}\right)\right]-\sum_{i=0}^{s^{2}}\sum_{j=0}^{B}1_{ij}^{noobj}\left[c_{ij}\log\left(\hat{c_{ij}}\right)+\left(1-c_{ij}\right)\log\left(1-\hat{c_{ij}}\right)\right],$$

where *S* ^2^ is scale of the feature map, *B* is the number of bounding boxes, $1_{ij}^{obj}\in \left\{0,\;1\right\}$ , if there is an object at the j-th bounding box of the i-th grid, the value is 1, otherwise the value is 0, $c_{ij}$ is the confidence of true value, $\hat{c_{ij}}$ is the confidence of predicted value.

The classification loss $l_{cls}$ is defined as follows:


(11) $$l_{cls}=-\sum_{i=0}^{s^{2}}\sum_{j=0}^{B}1_{ij}^{obj}\left[p_{ij}\log\left(\hat{p_{ij}}\right)+\left(1-p_{ij}\right)\text{log}\left(1-\hat{p_{ij}}\right)\right],$$

where $p_{ij}$ is the true probability of the target, $\left(\hat{p_{ij}}\right)$ predicted class probability of the target. The location loss $l_{loc}$ is defined as follows:


(12) $$l_{loc}=\sum_{i=0}^{s^{2}}\sum_{j=0}^{B}1_{ij}^{obj}\left[1-IoU+\left(\frac{\rho^{2}\left(b,\;b^{gt}\right)}{c^{2}}\right)+\alpha \nu \right],$$

where $\nu =\frac{4}{\pi^{2}}{\left(arctan\frac{w^{gt}}{h^{gt}}-arctan\frac{w}{h}\right)}^{2}$ ; $\alpha =\frac{\nu }{\left(1-IoU\right)+\nu }$ , $\rho \left(b,\;b^{gt}\right)$ is center distance between bounding box and ground truth box, c is the diagonal length of the smallest enclosing box covering two boxes.

In addition, focal loss, which proposed in RetinaNet and is shown in equation 13, is often used to solve the imbalance of sample categories and the imbalance of sample classification difficulty.


(13) $$L_{fl}=\{\begin{array}{c} -\alpha {\left(1-p\right)}^{\gamma }\log\left(p\right)\;\;\;\;,\;\;\;if\;y=1\\ -\left(1-\alpha \right)p^{\gamma }\log\left(1-p\right)\;,\;otherwise \end{array},$$

where p is the probability that the model predicts, y is GT class, α and γ are hyperparameters used to balance loss.

**Figure 5:**
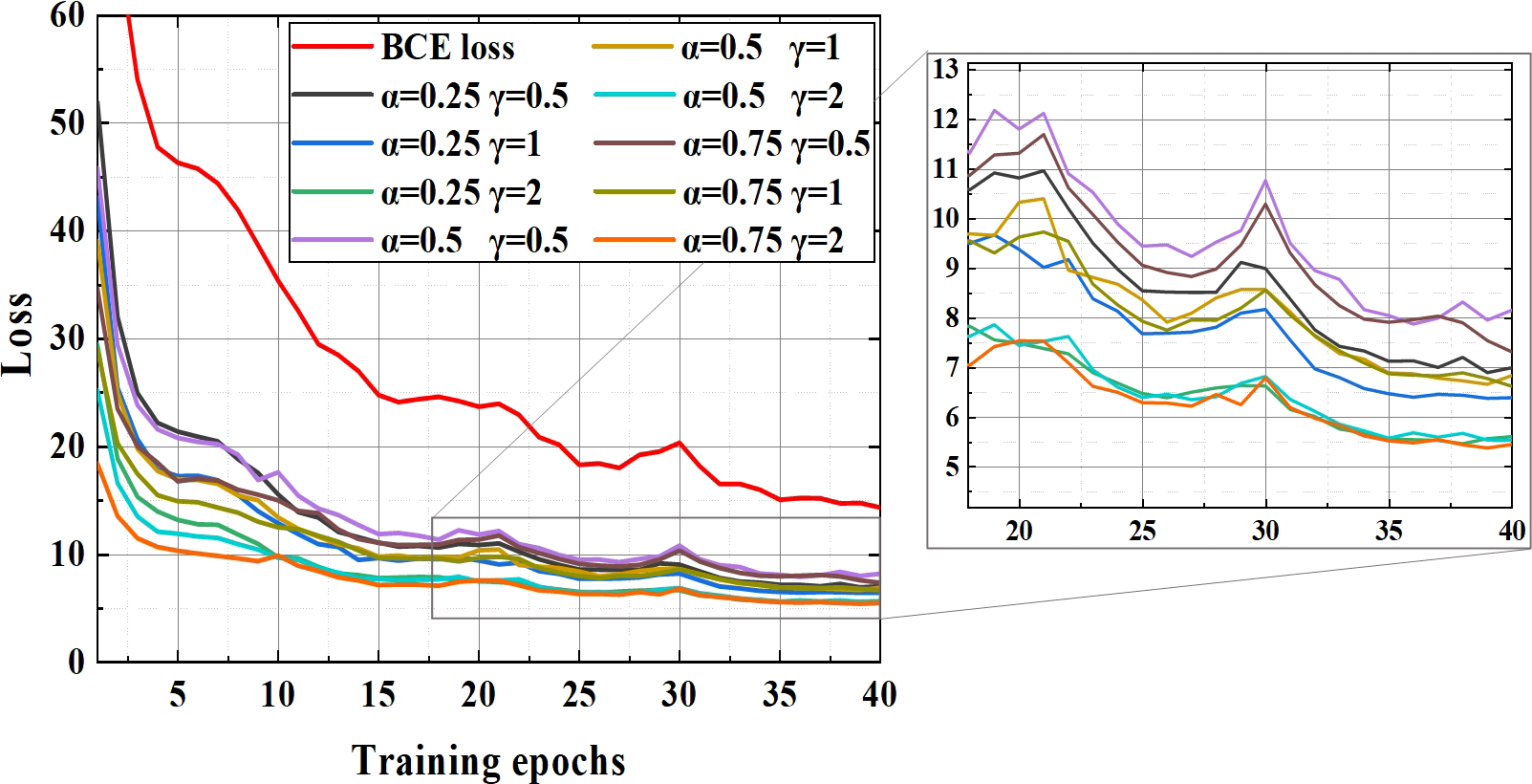
The curve of different hyperparameters schemes.

In order to explore whether should focal loss function be used to optimize the BCE loss function, the loss in different hyperparameter configurations were tested and these loss curves are shown in the **Figure 5**.

The downward trend of all curves is similar and the larger the value of hyperparameters, the faster the loss convergence speed and the lower the final saturation value. Reason for the fluctuations in **Figure 5** (epoch 30) is the use of the training strategy of freezing parameters.

The APs of YOLO v4+ model using different loss function schemes and the AP of the model without focal loss are shown in **Table 4**. Judging from the loss curves, all the effects of using focal loss seem to be better than only using the BCE loss function. However, it can be seen from **Table 4**. that only when the hyperparameters are set to α=0.75 and γ=2 , the AP of YOLO v4+ reaches 94.25%, which exceeds that of model without focal loss function. Therefore, in order to make the model has better performance, the optimal parameters should be determined according to different data sets, different target categories, etc.

**Table 4:** AP values with and without focal loss.

γ α	0.5	1	2	Only BCE loss function
0.25	91.7	91.91	90.88	93.85
0.5	93.27	92.85	93.21	
0.75	93.44	92.81	94.25	

## 4 Results and discussion

### 4.1 Detection performance of YOLO v4+ in unstructured environment

**Figure 6:**
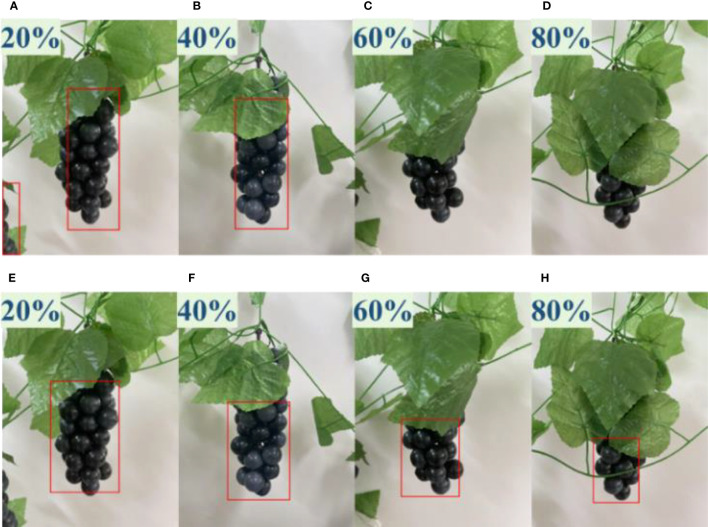
Comparative experiment on the detection performance of YOLO v4 and YOLO v4+ under different occlusion degrees. **(A–D)** Detection results of YOLO v4 with occlusion degrees of 20% to 80%, **(E–H)** Detection results of YOLO v4+ with occlusion degrees of 20% to 80%.

It is well known that grapes are grown in a typical unstructured environment. It is a common scenario for branches and leaves block the fruit, and grape bunches block each other. In fact, bunch occlusion does not affect visual recognition results, as the entire bunch on the front side is visible. The positions of the bunches can be determined by point clouds of two clusters of grape bunches located at different distances. Therefore, a quantitative comparative test experiment of the detection performance of YOLO v4 and the improved YOLO v4+ detector on leaves occlusion case was carried out. The sheltered area is 20 to 80 percent of the bunches. The experimental results are shown in **Figure 6**.

According to **Figure 6**, it can be seen that the YOLO v4 model missed detection when the occlusion reached 60% and 80%, while YOLO v4+ still successfully detected grapes. In addition, YOLO v4 also mistakenly identifies non regions of interest as positive samples when the occlusion is 20%.

**Figure 7:**
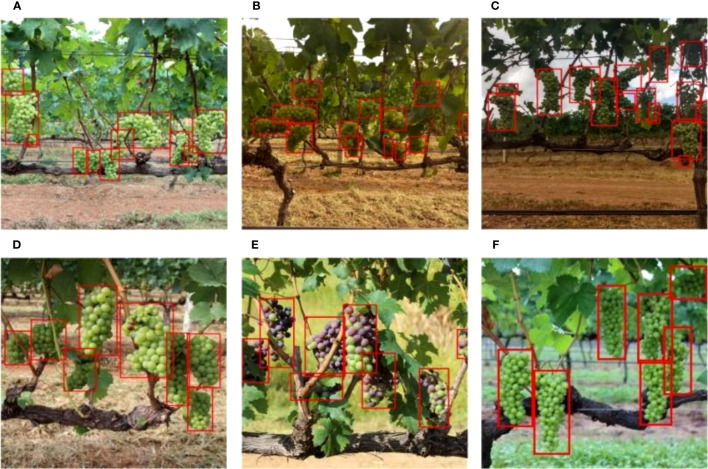
Detection results of YOLO v4+ in unstructured environment: **(A)** front-lighting, **(B)** backlighting, **(C)** insufficient light, **(D)** overlapping grapes, **(E)** occlusion by leaves, **(F)** inappropriate focus.

In addition, the fluctuation of illumination intensity and illumination angle cannot be ignored, and green grapes have a similar color to leaves, which are easy to cause interference. Verification experiments were carried out based on the YOLO v4+ model in the above several environments, and the detection results are shown in **Figure 7**.

**Figure 7A**, the color of the grapes becomes whitish under strong sunlight, causing the detection targets to lose the color characteristics. **Figure 7B** shows that opposite shooting direction also leads to color distortion. The grapes in **Figure 7C** were obtained under cloudy weather, and insufficient light makes the target dark. The overlapping grapes and occluded grapes in **Figures 7D, E** lose their complete contour. Inappropriate focus caused the blur of **Figure 7F**. And, most grapes are surrounded by green leaves. All grapes in these environments are identified by the proposed model.

These experimental results prove that the YOLO v4+ model has robustness to deal with the unstructured environment of grape growth. Changes in illumination intensity and angle, occlusion of fruits and overlap of fruits do not affect the accuracy of the proposed model to detect grapes.

### 4.2 Ablation experiments

This section demonstrates the effectiveness of data enhancement, SimAM, PAN+, and Focal loss in improving model performance through a series of ablation experiments. The AP and F1 scores for different methods are shown in **Table 5**. YOLO v4 without data enhancement is selected as the baseline, which is represented by A. According to Method B in **Table 5**, the performance of the model using data enhancement has significantly improved. When the SimAM module is embedded into Method B, the AP increases by more than 1%. Similarly, the AP of method B using the PAN+ has also been improved by 1.58%. Method E adopts data enhancement, while also integrating SimAM and PAN+ modules, resulting in an increase of 11.43% and 9% in AP and F1 compared to baseline, respectively. Method F further uses Focal loss, and AP has increased by 0.4% over Method E. The ablation experiments show that the methods used in this paper are consistent with expectations, which can more fully propagate features, and improve the robustness of the model to cope with occlusion and overlap.

**Table 5:** Performance of different detection models on grape dataset.

Methods	Data augmentation	SimAM	PAN+	Focal loss	AP [%]	F1 [%]
A	×	×	×	×	82.42	83
B	√	×	×	×	90.9	90
C	√	√	×	×	91.93	90
D	√	×	√	×	92.48	91
E	√	√	√	×	93.85	92
F	√	√	√	√	94.25	93

### 4.3 Comparison of different models in unstructured environment

In order to verify the superiority of the model proposed in this article, we compare YOLO v4+ with many SOTA detection models, such as SSD, Faster R-CNN, EfficientDet-D1, YOLO v3, YOLO v4, YOLO v7, YOLO v8 and CenterNet.

#### 4.3.1 Evaluation indicators of different models

**Figure 8:**
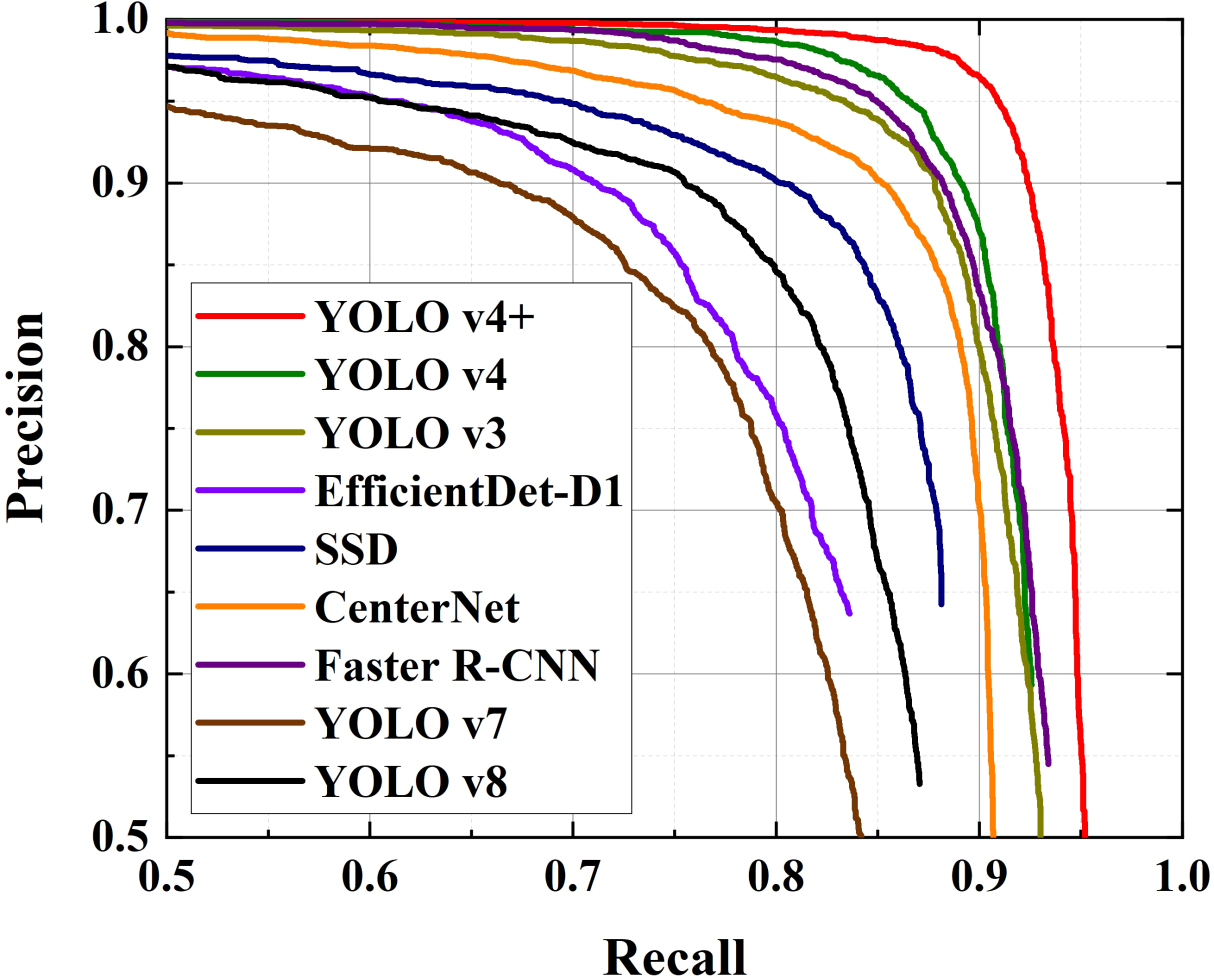
P-R curves for different detection methods.

In order to ensure the fairness of the comparison experiment, the input images’ size of most models is adjusted to 512×512. The input size marked by “–” represent that the scale of input image is randomly resized to 800^2^-1333^2^ pixels, which adopts the strategy of the official code. The size of the input image of EfficientDet is determined by its version. The P-R curves, AP and F1 score for these models generated by testing are presented in **Figure 8** and **Table 6** respectively.

**Table 6:** Performance of different detection models on grape dataset.

Model	Backbone	Input size [pixels]	AP [%]	F1 [%]
YOLO v4+	SAM-CSPDarkNet 53	512 × 512	94.25	93
Faster R-CNN	MobileNet V2	—	91.57	88
YOLO v3	DarkNet 53	512 × 512	91.06	89
YOLO v4	CSPDarkNet 53	512 × 512	90.90	90
CenterNet	ResNet 50	512 × 512	88.52	74
SSD	VGG 16	512 × 512	85.05	85
YOLO v8-s	C2f-CSPDarkNet	512 X 512	82.76	77
EfficientDet-D1	EfficientNet	640 × 640	79.53	80
YOLO v7-l	ELAN-CSPDarkNet	512 X 512	78.33	74

It can be clearly seen from **Figure 8** that the area enclosed by the red line (YOLO v4+) and the coordinate axis is the largest. Similarly, **Table 6** shows that the AP value of YOLO v4+ is the highest among several models, with a value of 94.25%, which is 3.35% higher than YOLO v4. And, YOLO v4 is comparable to YOLO v3 and Faster R-CNN in AP evaluation. There are obvious differences in the areas covered by the curves of the remaining models. Clearly, YOLO v7 covers the smallest area, followed by EfficientDet, YOLO v8, SSD, and then CenterNet. This result implies that in a natural orchard, the detection accuracy of the YOLO v4+ model is higher than other models. In other words, YOLO v4+ has higher reliability. Besides, what stands out in **Table 6** is that the F1 score of proposed method is 93%, which is 3% higher than other models with the highest scores. CenterNet and YOLO v7 got the lowest F1 score of 74%. F1 scores of EfficientDet and YOLO v8 are also unsatisfactory. As the latest models, the poor performance of YOLO v7 and YOLO v8 in grape detection in unstructured environment may be due to their improvements focus on the lightweight in order to pursue faster detection speed. The scores of the other three models are between 85% and 90%, which are relatively in the middle level with all experimental models. Based on the above results and analysis, it is shown that the comprehensive performance of the proposed model YOLO v4+ is better than the other SOTA models.

#### 4.3.2 Comparison of detection results under heavy fog

**Figure 9:**
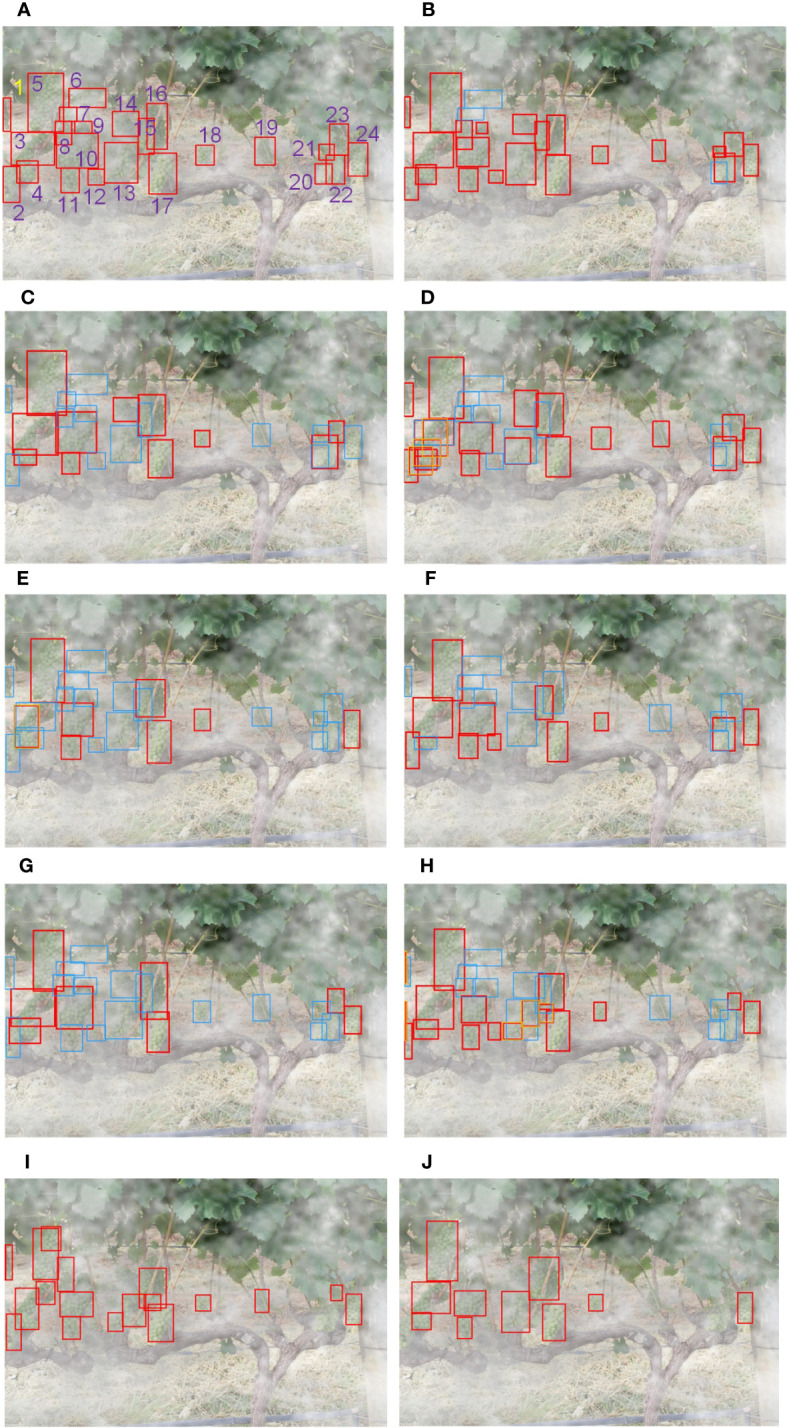
Detection results of grapes in a severely obstructed environment due to the heavy fog: **(A)** ground truth boxes, **(B)** YOLO v4+, **(C)** SSD, **(D)** Faster R-CNN, **(E)** EfficientDet-D1, **(F)** YOLO v3, **(G)** YOLO v4, **(H)** CenterNet, **(I)** YOLO v7-l, **(J)** YOLO v8-s, where grapes are TP in red boxes, FN in blue boxes, and FP in orange boxes.

Heavy fog is a common weather in our lives, and the heavy fog will obscure the fruit, making fruit detection more difficult. So, we randomly selected a picture from the dataset, which contains 24 bunches of grapes, and masked it by foggy weather transform. YOLO v4+ and the above-mentioned SOTA detector are used for grape identification in heavy fog weather. The test results are shown in **Figure 9**.

We also counted the TP, FP, and FN values of the selected image detected by each model, and calculate the Precision, Recall and F1 score according to equations 6 and 7. The statistical Table is shown in **Table 7**.

**Table 7:** Detection Precision and Recall of different models in foggy weather.

Model	TP	FP	FN	Precision [%]	Recall [%]	F1 score[%]
YOLO v4+	21	0	3	100.00	87.50	93.33
Faster R-CNN	16	3	8	84.21	66.67	74.42
SSD	11	0	13	100.00	45.83	62.86
YOLO v3	11	0	13	100.00	45.83	62.86
YOLO v8-s	10	0	14	100.00	41.67	58.82
CenterNet	12	5	12	70.59	50.00	58.54
YOLO v7-l	11	7	13	61.11	45.83	52.38
YOLO v4	8	0	16	100.00	33.33	50.00
EfficientDet-D1	7	1	17	87.50	29.17	43.75

In this situation, none of these models can achieve 100% F1 score. **Figure 9A** shows all 24 GT boxes and numbers them for the convenience of the following description. As can be seen from **Figure 9B**, the target grapes numbered 8, 9 and 21 are successfully detected by the exclusive model, which is YOLO v4+. And, only the proposed model and Faster R-CNN model correctly identified No.13 GT boxes. However, **Figure 9D** demonstrates that Faster R-CNN detected the grape numbered 13 with unsatisfactory Intersection over Union (IoU), and produced redundant bounding boxes on the detection of targets numbered 3 and 4. It should be noted that except for YOLO v4+, Faster R-CNN, and CenterNet, the Recall values of other models are all less than 50%. That is to say, the bunches of grapes identified by these models less than half of the true numbers. Fortunately, due to the rigorous detection mechanism, there was no mistaken detection in SSD, YOLO v3, YOLO v8 and YOLO v4. Unlike them, the proposed method also achieved 100% Precision with a Recall value of 87.5%. The other four models had false detections. From **Figure 9I**, we find that the number of grapes detected incorrectly by YOLO v7 is the most. In addition, due to the high concealment of GT boxes numbered 6, 7 and 20, they were missed by all the models in this experiment. Except for these three grapes, there are no additional missing targets in YOLO v4+.

Further, from **Table 7**, we can see that the number of TP detected by YOLO v4+ is 21, which is the most among all experimental models. Although the RPN in Faster R-CNN is specifically used for location, the number of correct detections is still 5 less than the proposed method. In addition, YOLO v7 and CenterNet have 7 and 5 false detections, respectively, which leads to a decline in their overall performance. As the basic model in this paper, YOLO v4 correctly identified 8 targets, which is 13 fewer than that identified by the proposed model. YOLO v4+ not only has relatively outstanding Recall, but also has 100% precision. The F1 score of YOLO v4+ is 93.33%, which is much higher than the other eight models. The F1 scores of SSD, YOLO v3 and YOLO v8 surpass that of CenterNet, which is benefit from the absence of mistaken detection in SSD, YOLOv3 and YOLO v8.

Compared with the other eight models, the model proposed in this article has better generalization ability, the detection accuracy in heavy fog weather is still the highest relative to other models. Specifically, the proposed method is better than other models in the recognition performance, especially in Recall. Moreover, all models except YOLO v4+ identified two clusters of grapes numbered 15 and 16 as a single target. This result firmly proves the superiority of the model proposed in this article for severe obscured fruit detection.

#### 4.3.3 Comparison of model parameters and detection speed

**Figure 10:**
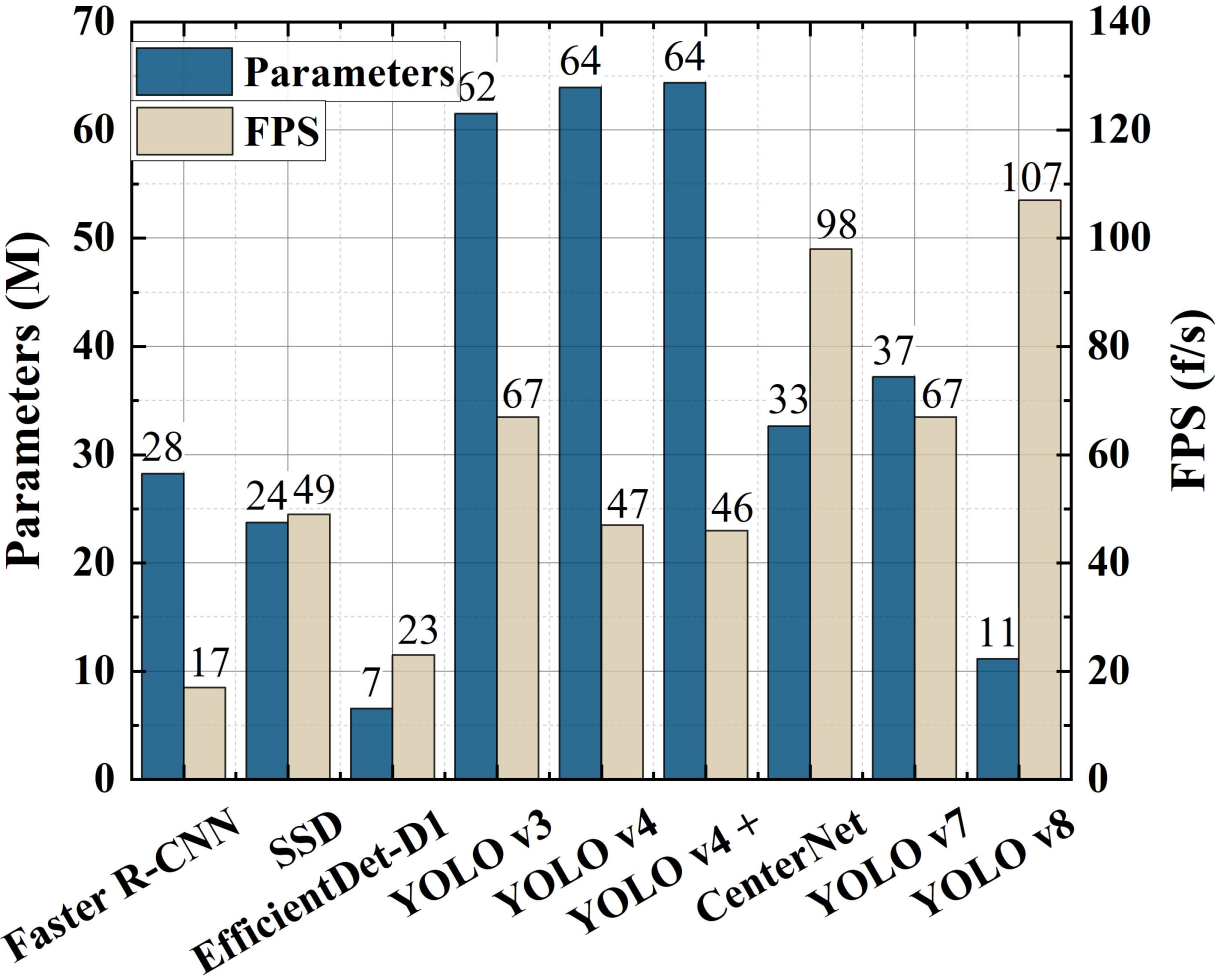
Comparison of parameters and detection speed between different models.

Since we embedded the SimAM block in the backbone and added an additional connection path in the neck, more computing resources may consume. In order to evaluate the model proposed in this article more comprehensively, we calculated the parameters and detection speed of these models and drew histograms as shown in **Figure 10**.

As we can see from histograms, compared to YOLO v4, the proposed model hardly increases the amount of parameters, and the detection speed is only reduced by 1 FPS, but the detection performance of YOLO v4+ significantly improved. It is worth noting that Faster R-CNN is two-stage model, so its detection speed is the slowest. Thanks to the depthwise separable convolution, the EfficientDet-D1 model has very few parameters, but still has a lower detection speed. The 640 × 640 input size determined by the EfficientDet version may have caused this result. Because the YOLO v4 model has a series of tricks mentioned in Section 1.1, it is normal that the number of parameters increases and the detection speed decreases compared to YOLO v3. There are fewer convolutional layers in backbone of SSD model, but the lack of optimization results in the loss of corresponding high detection speed. Both YOLO v8 and CenterNet use an anchor-free strategy to achieve the first and second fastest detection speed among several models. Surprisingly, YOLO v7 is lighter and should have higher detection speed compared to YOLOv3, but its performance is disappointing.

Since the model proposed in this article is an improvement of YOLO v4, even though the detection speed of proposed method is not the fastest in all models, we should focus on the difference between the model proposed in this article and YOLO v4. It is known from the experimental results that the model parameters and detection speed of YOLO v4+ are roughly the same as those of YOLO v4. Therefore, the embedding of the attention block and the newly-added connection path greatly improve the detection precision of the model without increasing the amount of calculation and hardly reducing the detection speed.

### 4.4 Impact of experimental dataset size

**Figure 11:**
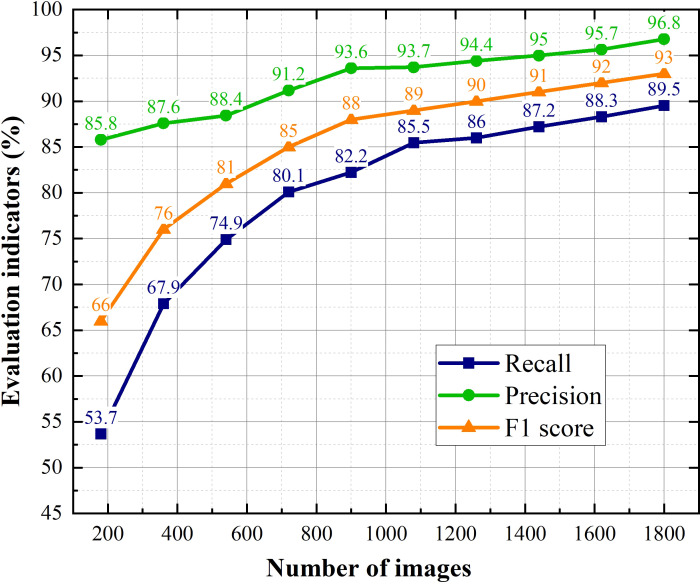
F1 score, P and R of YOLO v4+ trained with different size of datasets.

**Figure 12:**
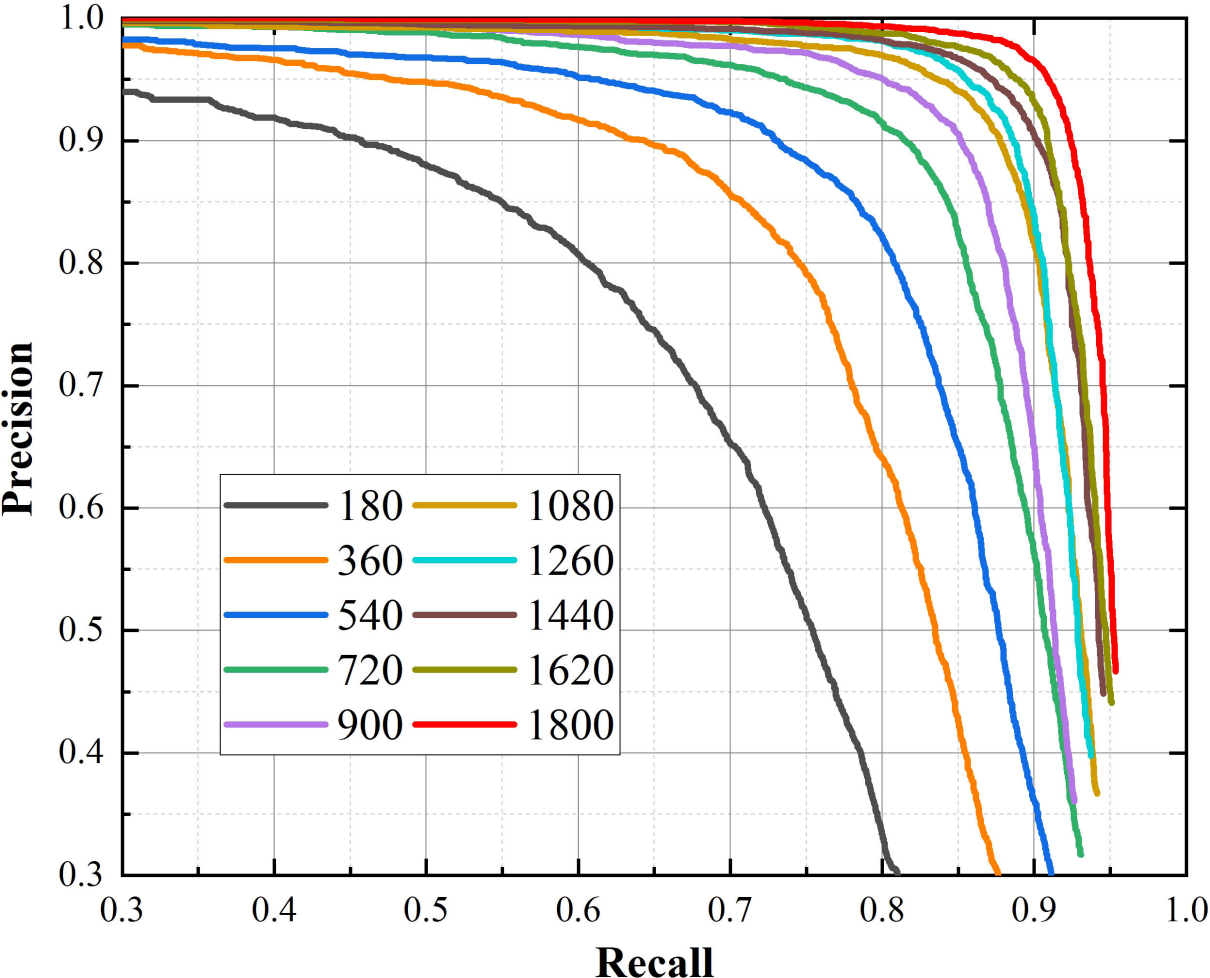
P-R curves of YOLO v4+ trained with different size of datasets.

The purpose of experiment in this section is to explore the impact of dataset on performance of the proposed model. We randomly selected 10%, 20%, 30%, 40%, 50%, 60%, 70%, 80%, 90%, 100% of the dataset, and obtained 10 datasets for experiments. The evaluation indicators and P-R curves for proposed method corresponding to different quantities of training images are shown in **Figures 11**, **12**.

The most obvious finding to emerge from **Figures 11**, **12** is that as the dataset expands, the performance of the model improves. The value of Recall rises from fast to slow when the number of images exceeds 1080, while the precision has been steadily increasing. This result means that Recall is more sensitive to the size of the dataset than precision. Because the F1 score integrates Recall and precision, it shows the same trend of change under the effect of Recall. When the amount of images in dataset exceeds 1080, the F1 score is gradually saturated, but the improvement of that is rapid before then.

### 4.5 Impact of data augmentation methods

The data augmentation methods selected in Section 3.2 can theoretically solve various disturbances. In order to verify the effectiveness of these methods, we removed one of the data augmentation methods each time and obtained the AP and final loss values as shown in **Table 8**.

**Table 8:** AP and saturated loss value for YOLO v4+ with removing different data augmentation method.

Data augmentation method	AP [%]	Saturated loss value
Dataset after augmentation	94.25	5.38
Remove raindrop processing	89.72	6.75
Remove mirror transformation	93.11	5.91
Remove affine transformation	93.23	5.84
Remove blur processing	92.87	6.85
Remove brightness transformation	92.72	6.13
No augmentation method	89.04	7.21

Raindrop processing is the same as we expected, it is extremely beneficial to improve the robustness of proposed method. The AP of the model trained with the dataset, which without raindrop processing, drops by 4.53%, and the saturated loss value rises to 6.75. Blur processing is favorable for the model to improve the detection accuracy of unclear images captured by the camera. The final sTable loss value is still 6.85, which means that the training effect of the model lacking blur processing is poor. Brightness transformation makes the model reliable to deal with the fluctuation of illumination throughout the day. The precision of the model after removing the brightness transformation drops to 92.72%. Mirror transformation and affine transformation, as methods to enrich the dataset, have a slight help for training the model. The performance of YOLO v4+ trained on the dataset without these two augmentation methods is worse than that of complete dataset.

From the experimental results, no matter which data enhancement method is removed, the AP of the proposed model decreases to varying degrees and the final convergence loss value increases significantly. The effectiveness of the augmentation methods chosen in this paper can also be proved by the comparison without using the augmentation methods and the complete dataset. Selecting the corresponding augmentation method for specific working condition will greatly improve the model detection accuracy.

### 4.6 Impact of convolution type and number of modules in our improved PAN

**Figure 13:**
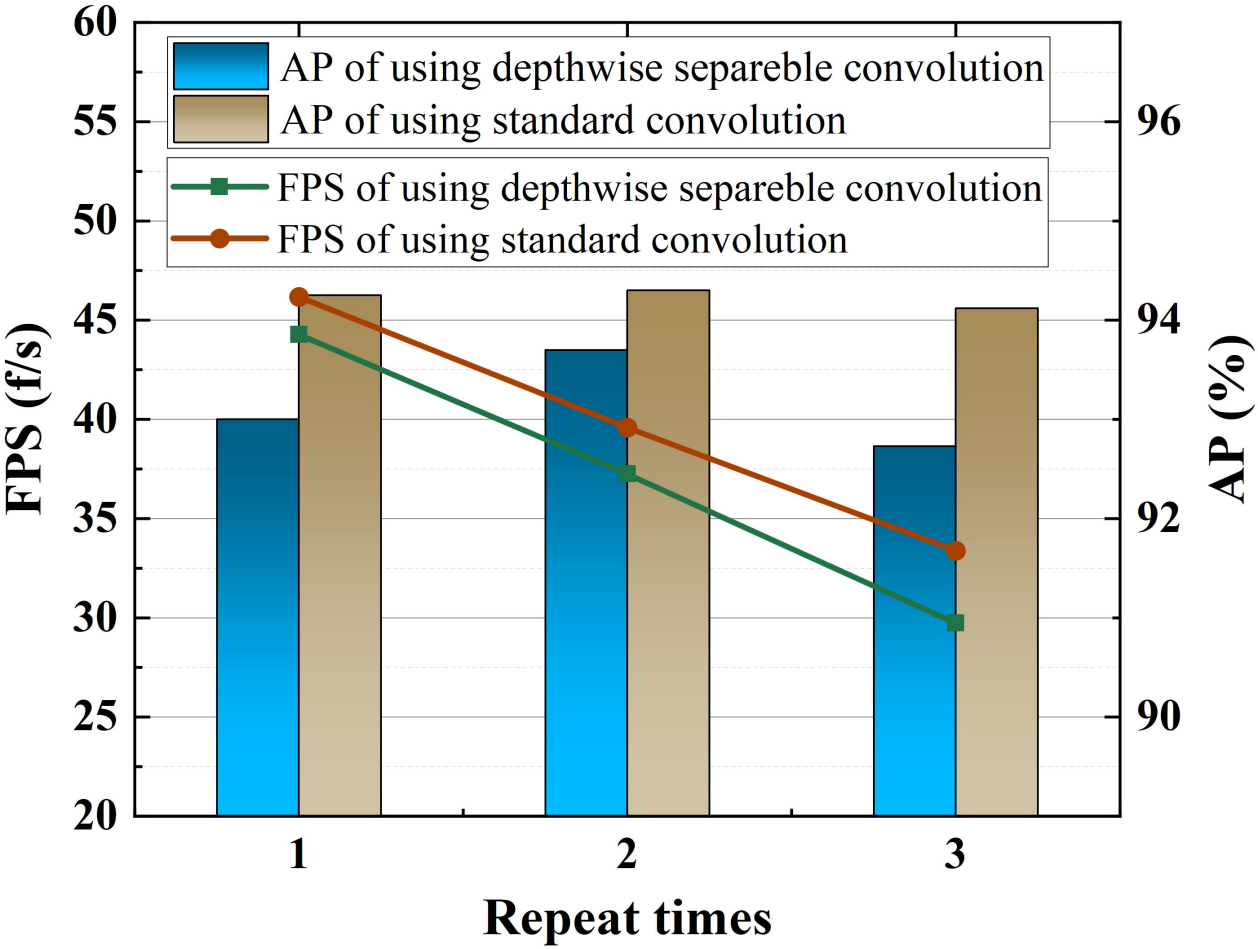
AP and FPS for different convolution types and module repetitions.

This experiment explains why our feature fusion module mentioned in Section 2.3 does not use depthwise separable convolution. It can be seen from **Figure 13** that with the increase of the number of repetitions of the feature fusion module, the FPS of both convolution types decreases linearly. However, no matter how many times the module is repeated, the AP of the model hardly changes. In addition, the models using standard convolution have better AP and FPS than the models using depthwise separable convolution. Therefore, we abandoned the depthwise separable convolution as a matter of course, and repeated the improved PAN once to save computing resources.

### 4.7 Instruction for YOLO v4+

**Figure 14:**
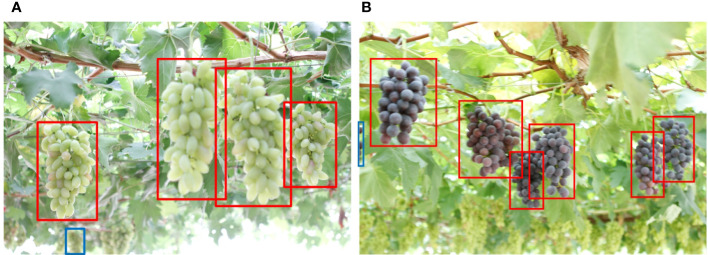
**(A, B)** Failure detection cases with YOLO v4+.

We took images from “Erya” Vineyard in Jurong, Jiangsu, China to verify the practical application of YOLO v4+. We found that the model is so sensitive to grape features that it detects object in the background that is not of interest, as shown in **Figure 14A**. However, this phenomenon does not work for all distant objects. Furthermore, **Figure 14B** demonstrates that grapes showing very little detail at the left edge of the image are also detected. Therefore, the harvesting robot system needs to judge the position and distance of the target when picking fruit.

## 5 Conclusion

This paper proposes a model for detecting grapes in unstructured environment, which is an improved version based on YOLO v4. SimAM attention blocks are embedded behind each feature extraction stage in CSPParkNet 53, and the innovative backbone called SAM-CSPDarkNet 53 is designed for feature extraction. Moreover, we refer to the connection structure of Bi-FPN in the EfficientDet model, and upgraded the feature fusion module of YOLO v4 using a weighted jump connection structure. In short, all adjustments are for the model to obtain comprehensive target feature pixels. We also use the focal loss function with hyperparameters α=0.75 and γ=2 to suppress the loss of massive negative samples. Ablation experiments have proven that each optimization method used in this paper is effective. The AP and F1 scores of YOLO v4+ are 94.25% and 93%, respectively.

Compared with several SOTA detection models, the above improvements make YOLO v4+ model has more excellent detection performance for severe occlusion, fruit overlap, illumination fluctuations, complex background and even the heavy fog weather without increasing the amount of calculation and hardly reducing the detection speed. Compared to YOLO v4, YOLO v4+ has an AP and F1 increase of 3.35% and 3%, respectively. Generally, YOLO v4+ not only has the highest comprehensive ability, but also has better generalization ability. Applying the proposed method to harvesting robots may enhance the applicability and robustness of the robotic system.

In addition, we found unexpectedly during the experiments that Recall is more sensitive to the size of the dataset than precision. In the case of insufficient datasets, augmentation methods can be used for data augmentation for a specific environment, such as cloudy and rainy weather. The depthwise separable convolution in YOLO v4+ does not improve FPS as expected, but reduces the AP.

In future work, we will (1) combine the point cloud information with the RGB of images to filter out objects beyond the robot workspace; (2) estimate the grape posture to improve grasping stability.

## Data availability statement

Publicly available datasets were analyzed in this study. This data can be found here: http://github.com/thsant/wgisd.

## Author contributions

CG: Formal analysis, Investigation, Methodology, Software, Validation, Writing - original draft, Writing - review & editing. SZ: Data curation, Project administration, Resources, Supervision, Writing - review &editing. GC: Project administration, Resources, Supervision, Writing - review &editing, Funding acquisition. YZ: Formal analysis, Investigation, Validation. JD: Project administration, Resources, Supervision. All authors contributed to the article and approved the submitted version.

## References

[B1] Bochkovskiy A. Wang C.-Y. Mark Liao H.-Y. (2020). YOLOv4: optimal speed and accuracy of object detection arXiv:2004.10934. doi: 10.48550/arXiv.2004.10934

[B2] Cai J. Tao J. Ma Y. Fan X. Cheng L. (2020). Fruit image recognition and classification method based on improved single shot multi-box detector. J. Physics: Conf. Ser. 1629 (1), 1–65. doi: 10.1088/1742-6596/1629/1/012010

[B3] Cai Z. Vasconcelos N. (2018). “Cascade r-CNN: delving into high quality object detection,” in Proceedings of the IEEE computer society conference on computer vision and pattern recognition (Salt Lake City, UT, USA: IEEE), 6154–6162. doi: 10.1109/CVPR.2018.00644

[B4] Carrasco M. (2011). Visual attention: the past 25 years. Vision Res. 51 (13), 1484–1525. doi: 10.1016/j.visres.2011.04.012 21549742 PMC3390154

[B5] Cecotti H. Rivera A. Farhadloo M. Pedroza M. A. (2020). Grape detection with convolutional neural networks. Expert Syst. Appl. 159, 113588. doi: 10.1016/j.eswa.2020.113588

[B6] Chaivivatrakul S. Dailey M. N. (2014). Texture-based fruit detection. Precis. Agric. 15 (6), 662–835. doi: 10.1007/s11119-014-9361-x

[B7] Darwin B. Dharmaraj P. Prince S. Popescu D. E. Hemanth D. J. (2021). Recognition of Bloom/Yield in crop images using deep learning models for smart agriculture: a review. Agronomy 11 (4), 1–225. doi: 10.3390/agronomy11040646

[B8] Girshick R. (2015). “Fast r-CNN,” in Proceedings of the IEEE international conference on computer vision (Santiago, Chile) 2015, 1440–1448. Inter. doi: 10.1109/ICCV.2015.169

[B9] Hu X. Liu Y. Zhao Z. Liu J. Yang X. Sun C.. (2021). “Real-time detection of uneaten feed pellets in underwater images for aquaculture using an improved YOLO-V4 network,” in Computers and electronics in agriculture, vol. 185. , 106135. doi: 10.1016/j.compag.2021.106135

[B10] Jocher G. (2023) New YOLO v8 in PyTorch. Available at: https://github.com/ultralytics/ultralytics.

[B11] Law H. Deng J. (2020). CornerNet: detecting objects as paired keypoints. Int. J. Comput. Vision 128 (3), 642–565. doi: 10.1007/s11263-019-01204-1

[B12] Lawal O. M. (2021). Development of tomato detection model for robotic platform using deep learning. Multimedia Tools Appl. 80 (17), 26751–26772. doi: 10.1007/s11042-021-10933-w

[B13] Li Y. He L. Jia J. Lv J. Chen J. Qiao X.. (2021). In-field tea shoot detection and 3D localization using an RGB-d camera. Comput. Electron. Agric. 185, 106149. doi: 10.1016/j.compag.2021.106149

[B14] Li G. Huang X. Ai J. Yi Z. Xie W. (2021). Lemon-YOLO: an efficient object detection method for lemons in the natural environment. IET Image Process. 15, 1998–2009. doi: 10.1049/ipr2.12171

[B15] Lin T. Yi Goyal P. Girshick R. He K. Dollar P. (2017). “Focal loss for dense object detection,” in Proceedings of the IEEE international conference on computer vision (Venice, Italy) , vol. 2017. , 2999–3007. doi: 10.1109/ICCV.2017.324

[B16] Lin G. Tang Y. Zou X. Cheng J. Xiong J. (2020). Fruit detection in natural environment using partial shape matching and probabilistic hough transform. Precis. Agric. 21 (1), 160–775. doi: 10.1007/s11119-019-09662-w

[B17] Liu W. Anguelov D. Erhan D. Szegedy C. Reed S. Fu C. Y.. (2016). “SSD: Single shot multibox detector,” in Lecture notes in computer science (Including subseries lecture notes in artificial intelligence and lecture notes in bioinformatics, vol. 9905. , LNCS:21–37. arXiv:1512.02325. doi: 10.1007/978-3-319-46448-0_2

[B18] Misra D. (2019). Mish: a self regularized non-monotonic activation function. arXiv 1908.08681. doi: 10.48550/arXiv.1908.08681

[B19] Moreira G. Magalhães S. A. Pinho T. Santos F. N. D. Cunha Mário (2022). Benchmark of deep learning and a proposed HSV colour space models for the detection and classification of greenhouse tomato. Agronomy 12 (2):356. doi: 10.3390/agronomy12020356

[B20] Nguyen H.-c. Nguyen T.-h. Scherer Rafał Le V.-H. (2023). YOLO series for human hand action detection and classification from egocentric videos. Sensors 23 (6), 3255. doi: 10.3390/s23063255 36991971 PMC10058182

[B21] Pang J. Chen K. Shi J. Feng H. Ouyang W. Lin D. (2019). “Libra R-CNN: towards balanced learning for object detection,” in Proceedings of the IEEE computer society conference on computer vision and pattern recognition, vol. 2019-June. (Long Beach, CA, USA: IEEE), 821–830. doi: 10.1109/CVPR.2019.00091

[B22] Payne A. B. Walsh K. B. Subedi P. P. Jarvis D. (2013). Estimation of mango crop yield using image analysis - segmentation method. Comput. Electron. Agric. 91, 57–64. doi: 10.1016/j.compag.2012.11.009

[B23] Pinheiro I. Moreira G. da Silva D. Queirós Magalhães S. Valente António Oliveira P. M.. (2023). Deep learning YOLO-based solution for grape bunch detection and assessment of biophysical lesions. Agronomy 13 (4), 1–235. doi: 10.3390/agronomy13041120

[B24] Ren S. He K. Girshick R. Sun J. (2017). Faster r-CNN: towards real-time object detection with region proposal networks. IEEE Trans. Pattern Anal. Mach. Intell. 39 (6), 1137–1495. doi: 10.1109/TPAMI.2016.2577031 27295650

[B25] Santos T. Angelov D. Buiani M. (2019). Embrapa wine grape instance segmentation dataset - embrapa WGISD. Available at: https://github.com/thsant/wgisd

[B26] Silwal A. Davidson J. R. Karkee M. Mo C. Zhang Q. Lewis K. (2017). Design, integration, and field evaluation of a robotic apple harvester. J. Field Robotics 34 (6), 1140–1595. doi: 10.1002/rob.21715

[B27] Song Z. Fu L. Wu J. Liu Z. Li R. Cui Y. (2019). Kiwifruit detection in field images using faster r-CNN with VGG16. IFAC-PapersOnLine 52, 76–81. doi: 10.1016/j.ifacol.2019.12.500

[B28] Sozzi M. Cantalamessa S. Cogato A. Kayad A. Marinello F. (2022). Automatic bunch detection in white grape varieties using YOLOv3, YOLOv4, and YOLOv5 deep learning algorithms. Agronomy 12, 319.

[B29] Su S. Chen R. Fang X. Zhu Y. Zhang T. Xu Z. (2022). A novel lightweight grape detection method. Agric. (Switzerland) 12 (9), 13645. doi: 10.3390/agriculture12091364

[B30] Tan M. Pang R. Le Q. V. (2020). “EfficientDet: scalable and efficient object detection,” in Proceedings of the IEEE computer society conference on computer vision and pattern recognition (Seattle, WA, USA) 10778–10787. doi: 10.1109/CVPR42600.2020.01079

[B31] Tian Z. Shen C. Chen H. He T. (2019). “FCOS: fully convolutional one-stage object detection,” in Proceedings of the IEEE international conference on computer vision, vol. 2019-Octob. (IEEE), 9626–9635. doi: 10.1109/ICCV.2019.00972

[B32] Webb B. S. Dhruv N. T. Solomon S. G. Tailby C. Lennie P. (2005). Early and late mechanisms of surround suppression in striate cortex of macaque. J. Neurosci. 25 (50), 11666–11755. doi: 10.1523/JNEUROSCI.3414-05.2005 16354925 PMC6726034

[B33] Woo S. Park J. Lee J-Y Kweon I. S. (2018). “CBAM: convolutional block attention module,” in Computer vision - ECCV 2018, (Munich, Germany) 3–19. Available at: https://link.springer.com/chapter/10.1007/978-3-030-01234-2_1#citeas.

[B34] Wu D. Lv S. Jiang M. Song H. (2020). Using channel pruning-based YOLO v4 deep learning algorithm for the real-time and accurate detection of apple flowers in natural environments. Comput. Electron. Agric. 178, 105742. doi: 10.1016/j.compag.2020.105742

[B35] Yang L. Zhang R.-Y. Li L. Xie X. (2021). SimAM: a simple, parameter-free attention module for convolutional neural networks. Proc. 38th Int. Conf. Mach. Learn. 139, 11863–11874. Available at: https://proceedings.mlr.press/v139/yang21o.html

[B36] Yin W. Wen H. Ning Z. Ye J. Dong Z. Luo L. (2021). Fruit detection and pose estimation for grape cluster–harvesting robot using binocular imagery based on deep neural networks. Front. Robotics AI 8. doi: 10.3389/frobt.2021.626989 PMC8259879 34239899

[B37] Zhang C. Ding H. Shi Q. Wang Y. (2022). Grape cluster real-time detection in complex natural scenes based on YOLOv5s deep learning network. Agric. (Switzerland) 12 (8):1242. doi: 10.3390/agriculture12081242

[B38] Zheng Z. Wang P. Liu W. Li J. Ye R. Ren D. (2020). “Distance-IoU loss: faster and better learning for bounding box regression,” in AAAI 2020 - 34th AAAI conference on artificial intelligence (Palo Alto, California, USA: AAAI Press) 12993. doi: 10.1609/aaai.v34i07.6999

[B39] Zhou X. Wang D. Krähenbühl P. (2019). Objects as points. arXiv 1904.07850. doi: 10.48550/arXiv.1904.07850

[B40] Zhu B. Chen C. Shen F. Savvides Z. (2020). “Soft anchor-point object detection” in Computer vision – ECCV 2020 (Glasgow, UK: Springer International Publishing), 91–107. doi: 10.1007/978-3-030-58545-7

